# Proteomic Analysis of ARID1A-Deficient Ovarian Clear Cell Carcinoma Cells Reveals Differential Mitochondria ETC Subunit Abundances and Targetable Mitochondrial Pathways

**DOI:** 10.3390/ijms26125466

**Published:** 2025-06-07

**Authors:** Jesenia M. Perez, Joohyun Ryu, Hannah Khan, Mihir Shetty, Emma Parker, Padraig D’Arcy, Shijia Zhu, Martina Bazzaro, Stefani N. Thomas

**Affiliations:** 1 Microbiology, Immunology, and Cancer Biology Graduate Program, University of Minnesota School of Medicine, Minneapolis, MN 55455, USA; 2 Department of Laboratory Medicine and Pathology, University of Minnesota School of Medicine, Minneapolis, MN 55455, USA; 3 Masonic Cancer Center and Department of Obstetrics, Gynecology and Women’s Health, University of Minnesota, Minneapolis, MN 55455, USA; 4 School of Biological, Health and Sports Sciences, Technological University Dublin, D07 EWV4 Dublin, Ireland; 5 Department of Biomedical and Clinical Sciences (BKV), Linköping University, 58183 Linköping, Sweden

**Keywords:** ARID1A, mitochondria, ovarian clear cell carcinoma, electron transport chain, proteomics

## Abstract

ARID1A-deficient ovarian clear cell carcinoma is a highly lethal gynecologic cancer that depends heavily on mitochondrial respiration. Our biochemical and proteomic analyses reveal that ARID1A knockout cells exhibit marked upregulation of specific subunits within mitochondrial electron transport chain (ETC) Complexes I, III, and IV. However, this upregulation does not directly translate into increased sensitivity to broad-spectrum inhibitors targeting these complexes. These findings suggest that broad-spectrum mitochondrial inhibitors may not be effective therapeutic options for ARID1A-deficient cancers. Instead, the selective inhibition of specific ETC subunits may offer a more promising approach to exploit the metabolic vulnerabilities of ARID1A-deficient cells.

## 1. Introduction

Ovarian clear cell carcinoma (OCCC) is a rare and deadly gynecological cancer characterized by inactivating mutations in *ARID1A*, a component of the SWI/SNF chromatin-remodeling complex, that occur in up to 70% of patients [1,2,3]. These patients have significantly worse prognoses, even when the cancer is diagnosed at early stages [4]. Thus, there is an unmet need for effective treatment modalities for *ARID1A*-mutated OCCC tumors.

Accumulating evidence suggests that components of the SWI/SNF chromatin-remodeling complex are critical regulators of mitochondrial respiration in mammalian cells, including ovarian cancer cells [5,6,7,8]. Therapeutics blocking mitochondrial respiration have been effective in other cancers including small cell lung cancer [9], prostate cancer [10], multiple myeloma [11], and breast [12]. In our recently published study, we evaluated the dependencies of OCCC-derived ARID1A wild-type and mutated cells on total mitochondria respiration [5]. We showed that a loss of ARID1A results in a higher dependency upon mitochondrial respiration corresponding to the upregulation of OXPHOS genes, and selective sensitivity to pan-mitochondrial inhibitors in vitro and in a preclinical xenograft model with ARID1A-mutated cell lines [5].

The results from these studies have contributed to the growing interest in targeting mitochondrial function for human cancer treatment. However, the clinical utility of the currently available FDA-approved drugs inhibiting mitochondrial function is hindered by the absence of precise data elucidating the ARID1A-associated expression patterns of individual components within the mitochondrial Electron Transport Chain (ETC). Not only are the currently available mitochondrial inhibitors used in non-stratified populations (i.e., in patients irrespective of their ovarian cancer subtype or mutational status), but they are also either pan-complex I ETC inhibitors (IACS-010759 or similar compounds) that cause side effects such as neuropathy, hyperlactatemia, and visual changes [13,14,15], or inhibitors that cause non-selective cell death (metformin) [16]. For these reasons, their use is associated with severe side effects and toxicity. These issues stem from their broad-spectrum activity and a lack of data on how ARID1A loss specifically affects the expression of individual components of the mitochondrial ETC.

To address these issues, in this study, we generated, characterized, and conducted a global proteomic analysis of ARID1A knockout clones from the OCCC-derived ovarian cancer cell line RMG1. In congruence with the results from our previously published study [5], the results from our global proteomic analysis demonstrate the significantly increased relative abundance of ETC proteins in the ARID1A knockout vs. wild-type RMG1 cells. Our data provide justification for identifying therapeutic vulnerabilities within the ETC in the context of treating ARID1A-deficient OCCC.

## 2. Materials and Methods

### 2.1. Chemicals and Antibodies

Rotenone (cat. # R8875), Antimycin A (cat. # A8674), KCN (cat. # 60178), Oligomycin (cat. # 75351) and Amido black stain 2× (cat. # A8181) were obtained from Millipore Sigma (Burlington, MA, USA). TTFA (Focus Biomolecules (Plymouth Meeting, PA, USA); # 10-4565-0100). Antibodies: anti-ARID1A (Millipore Sigma, cat. # HPA005456) and goat anti-rabbit-HRP (Cell Signaling Technology (Danvers, MA, USA), cat. #7074S). SuperSignal Substrate (Thermo Fisher Scientific (Waltham, MA, USA) cat. # 34095).

### 2.2. Generation of ARID1A Knockout (KO) Clones

Equimolar mixtures of two synthetic single guide RNAs were used (CGGGCGCAGAGTGCCATGGG and CAGGACCGCAGCAAGGACAT; CRISPRevolution sgRNA EZ Kit, Synthego (Redwood City, CA, USA); 1 or 2 µg in total), pre-complexed with 5 or 10 µg Cas9 protein (Alt-R^®^ S.p. Cas9 Nuclease V3, IDT (Newark, NJ, USA)) into ribonucleoproteins (RNPs). RNPs were introduced by electroporation of 0.5 or 1 × 10^6^ cells/100 µL tip using the Neon system (Thermo Fisher Scientific) with two 20 ms pulses and 1400 V in two subsequent rounds. Cells were expanded for several days prior to genomic DNA extraction (QIAamp mini kit, Qiagen (Hilden, Germany)). The frequency of edited alleles was estimated by droplet digital PCR (QX200 System, BioRad (Hercules, CA, USA). Single cells were sorted (Sony (Tokyo, Japan) SH800) into a 96-well plate to create single cell clones. For PCR confirmation, genomic DNA was extracted from the clones using the QIAamp DNA mini kit (Qiagen) and used for the PCR amplification of the edited locus using KAPA HiFi Hot start (Roche (Basel, Switzerland)) and specific oligos for ARID1A covering the edited region (FW 5′-gccatcaaagctcaggttaatg-3′ and REV 5′-catggaacggtgcctatagc-3′). PCR products were gel purified (Freeze N’ Squeeze, BioRad) and Sanger sequenced (Eurofins Genomics (Ebersberg, Germany)) to confirm ARID1A KO. Consistent disruption of both alleles was observed, confirming a homozygous knockout.

### 2.3. Cell Doubling Time

Cell counts were manually conducted at each cell line split using a hemocytometer. The total cell count was derived, and with the initial count (500,000 cells), the cell doubling time was calculated using the following formula: Doubling time = time (hours) × ln(2)/ln(final cell count/initial cell count). Calculated doubling times were then imported into GraphPad Prism (v8.0) to determine the average doubling time for each cell line.

### 2.4. Mitochondrial Labeling and Quantification of Mitochondrial Membrane Potential

Cells were stained with MitoTracker Deep Red FM dye (Cell Signaling Technology) according to the manufacturer’s instructions. Briefly, 500 nM MitoTracker Deep Red dye solution was added to cells growing on glass coverslips and incubated for 30 min at 37 °C. After uptake, the medium was removed, cells were fixed in ice-cold, 100% methanol for 15 min at −20 °C and rinsed 3 times with PBS for 5 min. Cells were mounted using ProLong Diamond Antifade with DAPI (Thermo Fisher Scientific). Images were acquired with a Zeiss LSM 700 confocal microscope using a 40× oil immersion objective. Fluorescence intensity quantification was performed for each condition using ImageJ2 open platform software with background subtraction.

### 2.5. Cell Viability Assay

For residual cell viability assay, 50,000 cells were plated/well of 24-well plates the day before being treated with the indicated drugs at the indicated concentrations over a period of 48 h. At the end of the treatment, wells were washed twice with PBS and the residual cells were trypsinized. Their viability and number were evaluated under microscopy via trypan blue exclusion. The calculation of relative survival (%) was performed by comparing the number of cells present in each treatment condition to the number of cells present in the untreated control condition (set at 100%). The assay was conducted in complete media containing a physiological concentration of glucose (5 mM glucose). IC_50_ values and graphical representation of data were performed using GraphPad Prism (version 10.1.1, GraphPad Software, San Jose, CA, USA). *n* = 3 for all experiments.

### 2.6. Western Blot

Cells were harvested and lysed in ice-cold lysis buffer (1% SDS in PBS containing 1× protease inhibitor cocktail) by probe sonication (20% pulse for 10 s) followed by centrifugation at 16,000× *g* for 20 min at 4 °C. The supernatant was run on 4–15% SDS-PAGE gels. Proteins were then transferred to nitrocellulose membranes and incubated at room temperature in 5% milk in 1× PBST buffer for 1 h. Membranes were then incubated overnight with primary antibodies at a dilution of 1:1000 in 1× PBST buffer. The next day, membranes were washed with 1× PBST buffer and incubated with anti-rabbit or anti-mouse secondary antibodies at a 1:2000 dilution for 1 h. Immunoreactive bands were detected by the FluorChem Fluorescent Western Imaging System from ProteinSimple (San Jose, CA, USA).

### 2.7. Protein Extraction and Enzymatic Digestion for Proteomic Analysis

RMG1 wild-type (WT) cells and ARID1A knockout clones (D9 and G11) were cultured in 60 mm × 15 mm plates (WT, *n* = 3; D9 clone, *n* = 3; G11 clone, *n* = 2), harvested upon reaching ~80% confluence by scraping and washed twice with cold PBS. After washing, the cell pellets were resuspended in 100 µL of lysis buffer (8 M urea, 75 mM NaCl, 50 mM Tris-HCl pH 8.0, and 1 mM EDTA) and subjected to sonication in an ice slurry. The cell lysates were clarified by centrifugation at 13,000 rpm for 20 min at 4 °C. Protein concentration was determined by a Pierce 660 nm Protein Assay Reagent (Thermo Fisher Scientific). An aliquot of 25 µg protein was prepared from each sample and used for enzymatic digestion. Reduction was carried out with 5 mM dithiothreitol for 1 h at 37 °C and alkylation was conducted using 10 mM iodoacetamide for 45 min at 25 °C. The samples were diluted 1:3.5 (*v*/*v*) with 50 mM Tris-HCl (pH 8.0). The samples were first digested with LysC (Wako (Zug, Switzerland)) at an enzyme/substrate ratio of 1 mAU/50 µg total protein for 2 h at 25 °C, followed by digestion with trypsin (Promega (Singapore)) at an enzyme/substrate ratio of 1:50 (*w*/*w*) for 18 h at 25 °C. The digestion reaction was quenched by acidification to a final concentration of 1% (*v*/*v*) formic acid (Thermo Fisher Scientific). The digested samples were then desalted using 1 mg C18 Sep-Pak cartridges (Waters, (Milford, MA, USA)). The eluted peptides were concentrated by vacuum evaporation in a Speed-Vac.

### 2.8. Basic Reversed-Phase Liquid Chromatography

The dried TMT-labeled peptides were reconstituted in 2% acetonitrile/4.5 mM ammonium formate by sonication in an ultrasonic water bath for 3 min and the insoluble component was pelleted by centrifugation at 13,000 rpm for 3 min at RT. Subsequently, peptide fractionation by basic reversed-phase liquid chromatography was performed using a 1200 Infinity II liquid chromatography system equipped with a ZORBAX 300 Extend-C18 column (length, 100 mm; inner diameter 2.1 µm; particle size 3.5 µm, flow rate, 200 µL/min) (Agilent Technologies (Santa Clara, CA, USA)). Mobile phases A and B contained 2% acetonitrile/4.5 mM ammonium formate in water and 90% acetonitrile/4.5 mM ammonium formate, respectively. Peptides were separated using a linear gradient as follows: 0–16% B for 6 min, 16–40% B for 60 min, 40–44% B for 4 min, and 44–60% B for 5 min. Fractions were collected at 1 min intervals and concatenated into 12 fractions, followed by freezing for 30 min at −80 °C and then concentrated by vacuum evaporation in a Speed-Vac. Prior to LC-MS/MS analysis, the peptides were reconstituted in 12 µL of 3% acetonitrile/0.1% formic acid and the peptide concentration was measured using a NanoDrop spectrophotometer (Thermo Fisher Scientific).

### 2.9. TMT Labeling

The dried peptides were reconstituted in 40 µL of 50 mM HEPES (pH 8.5) by sonication in an ultrasonic water bath for 3 min and the insoluble material was pelleted by centrifugation at 13,000 rpm for 3 min at RT. An equivalent amount of peptide from each sample was labeled with TMT 10 plex Mass Tag Labeling Reagents (Thermo Fisher Scientific) according to the manufacturer’s instructions. Briefly, 20 µg/µL of each TMT reagent was added at a TMT–peptide ratio of 7.5:1 (*w*/*w*). Each sample was labeled as WT replicate 1 (127C), WT replicate 2 (131), WT replicate 3 (130C), D9 clone replicate 1 (129C), D9 clone replicate 2 (128C), D9 clone replicate 3 (129N), G11 clone replicate 1 (128N), and G11 clone replicate 2 (130N). After confirming that the TMT labeling efficiency was >99% for each sample, all individual TMT reactions were quenched by adding 5% hydroxylamine (*v*/*v*) to a final concentration of 0.2% (*v*/*v*). Then, an equal amount of each TMT-labeled sample was combined, dried and desalted using C18 STAGE Tips. The desalted, TMT-labeled peptides were concentrated by vacuum evaporation in a Speed-Vac.

### 2.10. LC-MS/MS Data Acquisition

NanoLC-ESI MS/MS analysis was performed using a Q Exactive Plus hybrid Quadrupole-Orbitrap mass spectrometer coupled with an Ultimate 3000 RSLCnano HPLC system by an EASY-Spray Source, and an EASY-Spray C18 LC column (150 mm × 75 µm; 3 µm particles) with an integrated emitter and a PepMap C18 precolumn (5 mm × 0.3 µm; 5 µm particles) (Thermo Fisher Scientific). Mobile phases A and B contained 3% acetonitrile/0.1% formic acid in water and 90% acetonitrile/0.1% formic acid, respectively. Peptides (750 ng) were loaded on the PepMap C18 precolumn for 5 min at 3% mobile phase B (flow rate: 5 µL/min) and separated on the EASY-Spray LC column with a linear gradient of 3–40% mobile phase B for 90 min followed by 40–95% B for 2 min and 95% B for 5 min (flow rate: 300 nL/min).

The mass spectrometer was operated in a data-dependent acquisition mode. The parameter settings were as follows: mass range *m*/*z* 375–1400, resolution 70,000 at *m*/*z* 200, AGC target 3 × 10^6^, maximum injection time 50 ms for full MS scans; resolution 35,000 at *m*/*z* 200, AGC target 1 × 10^5^, maximum injection time 100 ms, isolation window 1.2 *m*/*z*, normalized collision energy 32, loop count 12, dynamic exclusion 30 s. The tune parameters were as follows: spray voltage 1.7 kV and capillary temperature 275 °C. The mass spectrometry data files have been deposited to the ProteomeXchange Consortium via the PRIDE [17] partner repository with the dataset identifier PXD050332. 

### 2.11. Mass Spectrometry Data Analysis

Raw mass spectrometry data were processed using MaxQuant version 2.1.4. Raw files were processed by setting group-specific parameters for enzymatic digestion, variable modifications, fixed modifications, and reporter ion MS2. Enzymatic digestion was set to specific with Trypsin/P and LysC with an allowable max number of missed cleavages set at 2. The oxidation of methionine and acetylation of protein N-terminus were set as variable modifications, and cysteine carbamidomethylation was set as a fixed modification. For TMT-labeled protein quantification, reporter ion MS2 was selected and 8 isobaric labels with their correction factors were applied from a TMT 10-plex set: 127C, 128N, 128C, 129N, 129C, 130N, 130C, 131N. All other parameters within MaxQuant remained under default settings. A *Homo sapiens* Swiss-Prot database (downloaded on 13 November 2023) was used for protein identification. All subsequent data analyses were performed using Perseus version 2.0 and RStudio (2024.04). Data from the ProteinGroups.txt file output from the database search conducted using MaxQuant were subsequently filtered and prepared using Perseus. Proteins only identified by a modification site, proteins identified as a potential contaminant from the CRAPome database, and proteins identified by their reverse sequence (decoys) were removed from the dataset. Data were log_2_ transformed followed by median normalization, and non-valid values were filtered out of the dataset. Outliers were identified by Grubbs’ test (*n* = 196) and subsequently removed from the dataset. G11 was omitted from downstream data analysis. The resulting dataset included 3316 quantified proteins identified across all samples.

### 2.12. Bioinformatic Analysis of Proteomic Data

To identify significantly dysregulated proteins between ARID1A clone D9 and WT RMG1 cells, protein abundances were analyzed using a two-sample T-test. Dysregulated proteins were identified by their level of significance (FDR < 0.01). The pathway analysis of significantly dysregulated proteins was conducted using the PANTHER Overrepresentation Test via the PANTHER knowledgebase v. 19.0. Fisher’s Exact test was conducted and Bonferroni correction was applied for multiple testing.

## 3. Results

### 3.1. Generation and Characterization of ARID1A Knockout (KO) Clones for Proteomic Analysis

We successfully generated stable CRISPR-Cas9-mediated ARID1A knockout (KO) clones from the well-characterized RMG1 OCCC cell line and evaluated their knockdown efficiency and growth rates. Both the D9 and G11 clones exhibited efficient ARID1A knockdown (Figure S1A,B) without affecting their doubling times (Figure S1C). For our subsequent analyses, we chose to use the D9 clone due to its superior knockdown efficiency.

**Figure 1:**
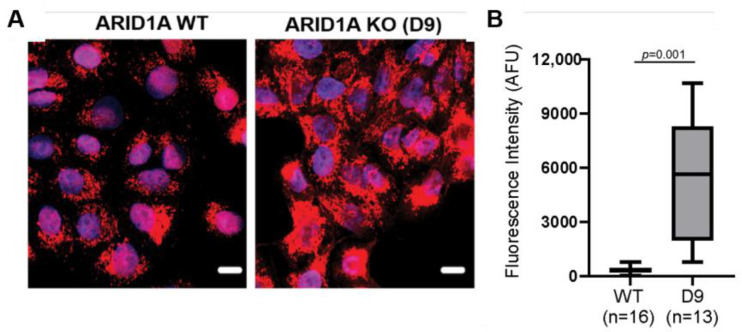
ARID1A KO cells have a higher mitochondrial potential compared to WT cells. (**A**) Representative merged images of WT and D9 clones stained with DAPI (blue) and Mitotracker Deep Red FM (red). Cells were maintained at low cell density to prevent confluence. Scale bar is 10 µm. (**B**) Quantification of Mitotracker Deep Red FM fluorescence intensity per condition expressed as arbitrary fluorescence units (AFU). *n* = number of cells evaluated per condition. Error bars indicate mean ± S.D. Student’s *t*-test.

Using this D9 clone, we confirmed our earlier findings [5] that the loss of ARID1A results in increased mitochondrial membrane potential as indicated by the greater accumulation of the Mitotracker Deep Red FM dye [18] in the D9 clones as compared to WT cells (*p* = 0.001) (Figure 1). This renders the D9 clones suitable for global proteomic analysis.

### 3.2. Global OCCC Proteome Profile of Loss of ARID1A Expression Reveals an Increased Relative Abundance of Mitochondrial Proteins

To obtain an unbiased assessment of the biological process perturbations associated with ARID1A loss, we conducted a moderate-depth global proteomic assessment of the D9 clone. A total of 3316 proteins were quantified without any missing values across all samples (Figure S3A) and with very strong correlations among the biological replicates (r ≥ 0.99; *n* = 3) (Table S1 and Figure S2). Hierarchical clustering of the 701 differentially expressed proteins (Figure S3B) and Principal Component Analysis (Figure S3C) revealed that the proteome of the D9 clone was markedly dissimilar from the proteome of the ARID1A WT cells.

**Figure 2:**
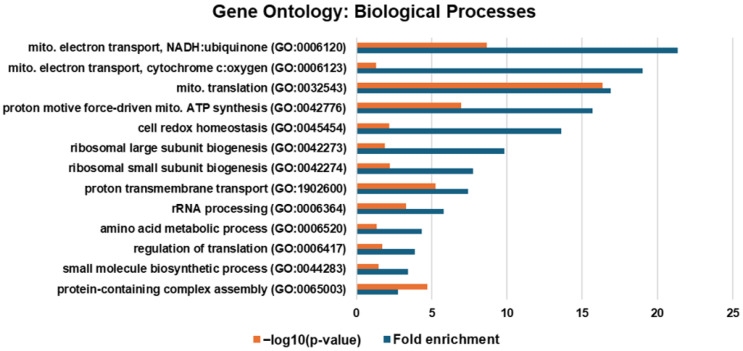
Gene Ontology Biological Process enrichment of proteins with increased relative abundance following ARID1A KO.

To identify significantly enriched pathways and protein–protein interaction networks associated with the lack of ARID1A expression, we conducted Gene Ontology (GO) pathway analysis using PANTHER with the 349 proteins having significantly increased abundance in the D9 clones. The top five Biological Processes with the greatest fold-enrichment were mitochondrial electron transport, mitochondrial translation, proton motive force-driven mitochondrial ATP synthesis, cell redox homeostasis, and ribosomal subunit biogenesis (Figure 2). The Biological Process with the highest fold-enrichment and significance was mitochondrial translation. The significant enrichment of the mitochondria-related pathways confirms the results from our differential protein abundance analysis. These results also corroborate the insight gained from a CRISPR screen of an ARID1A KO HEK293 cell line and an ARID1A KO RMG1 cell line, implicating the enrichment of mitochondrial respiratory chain complex assembly, NADH dehydrogenase complex assembly, and nucleoside monophosphate metabolic process Biological Processes [19,20].

The significant enrichment of ribosome biogenesis, amino acid metabolic process, and protein-containing complex assembly are novel findings, indicating putative targetable pathways for the treatment of OCCC characterized by the loss of ARID1A expression. Although they were not the focus of our current analysis, the significantly enriched biological pathways among the downregulated proteins included the regulation of macroautophagy and endosome organization. The downregulation of these pathways as a result of the loss of ARID1A expression indicates the perturbation of intracellular signaling.

**Figure 3:**
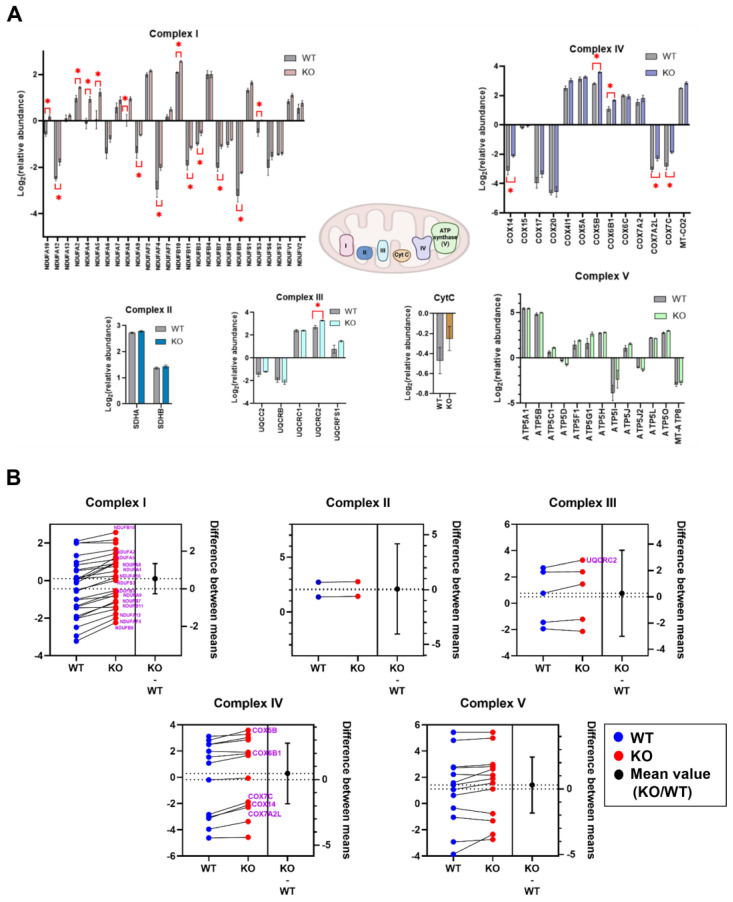
Differential expression of mitochondrial ETC components at the level of individual proteins vs. entire complexes. (**A**) Relative abundance of mitochondrial ETC proteins in the ARID1A WT vs. KO cells. * *p* < 0.05 (Welch’s *t*-test). Created in BioRender.com. (**B**) Estimation plots indicating the magnitude of differences in relative abundance between the ETC proteins. ETC proteins with significant differences in relative abundance between the ARID1A WT vs. KO cells are listed in purple font and are the same as those indicated in panel (**A**). The dashed horizontal lines indicate the lower and upper values of the 95% confidence interval. Differences between the mean protein abundance of each complex are plotted to the right of the solid vertical line. * *p* < 0.05.

Among the 3316 quantified proteins in the global proteome analysis, 499 were of mitochondrial origin based on the comparison with the mitochondrial high-confidence proteome (MitoCoP) database [21] (Figure S3D). To gain insight into ARID1A expression-related differences in mitochondrial proteins, we analyzed and quantified the detectable components of all five complexes of the ETC (Figure 3A). Among the proteins forming Complex I/NADH-ubiquinone oxidoreductase, the largest multi-subunit enzyme complex in the ETC, 14 had significantly increased relative abundance in the absence of ARID1A expression. Significantly increased relative abundances of cytochrome b-c1 complex subunit 2/Complex III and five cytochrome c oxidase subunits within Complex IV were observed in the D9 clones. Notably, when analyzed at the ETC complex level, significantly increased relative abundances of Complexes I, IV, and V were associated with ARID1A loss (Figure 3B). Taken together, increased relative abundances were observed at the individual subunit level for Complex III, but these differences were not re-capitulated at the level of the entire complex. Conversely, differences in the relative abundance of Complex V were not re-capitulated at the level of individual subunits.

### 3.3. ARID1A KO Results in Increased Sensitivity to Complex I and Complex V Mitochondrial Inhibitors

**Figure 4:**
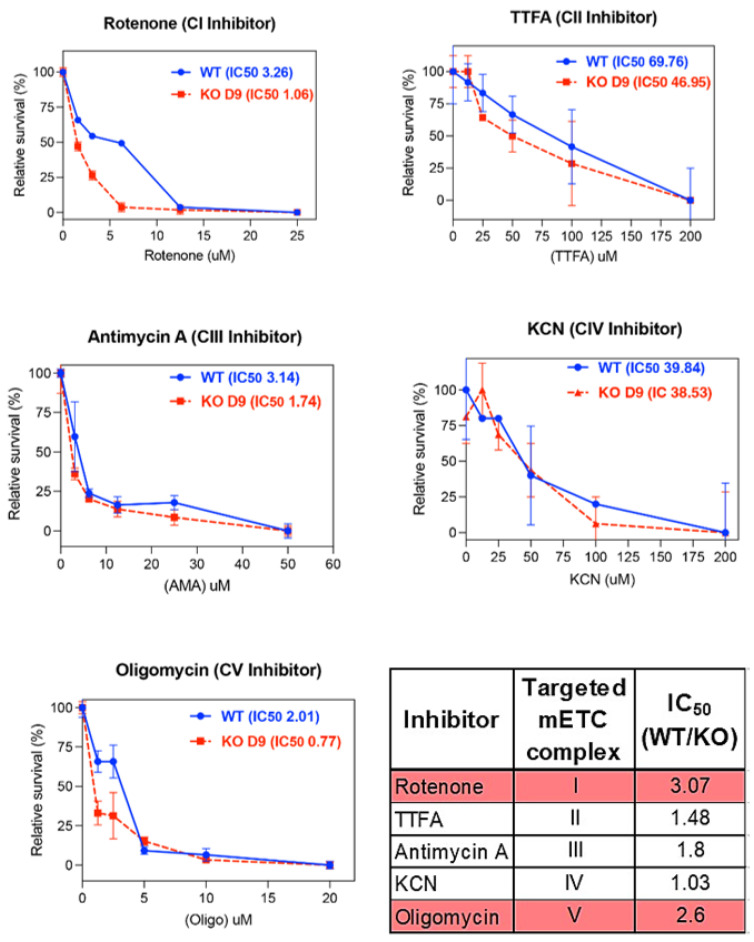
ARID1A KO selectively increases sensitivity to ETC complex I and V inhibitors. Dose-dependent sensitivity of OCCC-derived RMG1 ARID1A WT and ARID1A D9 clone to a panel of mitochondrial ETC CI-CV inhibitors. Results are expressed as residual cell viability as compared to control. Drug treatment was conducted over a period of 48 h. *n* = 3 replicates.

To gain insights into the effect of ARID1A loss on selective sensitivity to the currently available mitochondrial inhibitors, WT and D9 clones were exposed to increasing concentrations of each of the commercially available inhibitors of the mitochondrial Complex I (CI) inhibitor Rotenone [22], the Complex II (CII) inhibitor thenoyltrifluoroacetone (TTFA) [23], the Complex III (CIII) inhibitor Antimycin A [24], the Complex IV (CIV) inhibitor KCN [25], and the Complex V (CV) inhibitor Oligomycin [26] over time. The cell viability assay results indicated that the D9 clone exhibited selective sensitivity to inhibitors targeting complexes CI and CV with >2-fold differences in IC_50_ values, whereas their sensitivity to other inhibitors remained comparable to the WT clones (Figure 4). These results confirm our earlier observations, underscoring the importance of CI in the heightened reliance of ARID1A-mutated cells on mitochondrial respiration and also highlight CV as an additional potential therapeutic target.

## 4. Discussion

Our proteomic analysis showed that ARID1A loss results in increased levels of individual subunits from ETC Complexes I, III, and IV. When the proteomic data were analyzed at the entire Complex level, ARID1A loss was associated with increased levels of Complexes I, IV, and V. However, functional assays revealed that these cells are only sensitive to pan-inhibitors of Complex I and V—not III or IV. This disconnect highlights the need to interpret protein abundance data alongside functional assays when identifying drug targets. Broad ETC inhibition may not be effective for ARID1A-deficient cancers, but targeting specific subunits tied to altered metabolic dependencies may offer a more precise therapeutic strategy.

Taken together, our data indicate that OCCC cells upregulate ETC components as a compensatory mechanism of survival in the absence of ARID1A expression, which can be exploited as a therapeutic vulnerability. Our ongoing studies are designed to validate these findings by evaluating the mitochondrial proteomes of ARID1A WT and KO OCCC cell lines to enhance our ability to discern ETC component-specific perturbations. To address the in vitro limitation of our current study, we are expanding our analysis to the in vivo setting by analyzing the mitochondrial proteomes of patient-derived OCCC tumor tissue. The limitation of incomplete coverage of ETC Complex components will be addressed in future studies by adopting a targeted proteomic approach using mitochondrial fractions isolated by subcellular fractionation.

The long-term goal of our studies is to uncover novel therapeutic targets in ARID1A-deficient OCCC tumors. ARID1A mutations also occur in common cancers (e.g., colon, endometrial); thus, our findings in OCCC cells could have broader clinical significance. Given that increased ETC protein expression is a predictor of chemotherapy resistance in other subtypes of ovarian cancer [27], the impact of our findings could potentially be extended to the identification of novel therapeutic targets for chemo-resistant tumors.

## Data Availability

The mass spectrometry data files have been deposited at the ProteomeXchange Consortium via the PRIDE [17] partner repository with the dataset identifier PXD050332.

## Supplementary Materials

The following supporting information can be downloaded at https://www.mdpi.com/article/10.3390/ijms26125466/s1.

## Abbreviations

ACN, acetonitrile; CI-V, Complex I–V; ETC, electron transport chain; FDR, false discovery rate; KCN, potassium cyanide; KO, knockout; OCCC, Ovarian clear cell carcinoma; TMT, tandem mass tags; TTFA, thenoyltrifluoroacetone; WT, wild-type.
