# Supplementary material for: Proteomic Analysis of ARID1A-Deficient Ovarian Clear Cell Carcinoma Cells Reveals Differential Mitochondria ETC Subunit Abundances and Targetable Mitochondrial Pathways
Source: Int J Mol Sci. 2025 Jun 7;26(12):5466. doi: 10.3390/ijms26125466 (PMC12193532; doi:10.3390/ijms26125466)
Supplement: Supplementary file 1 [file ijms-26-05466-s001.zip › ARID1A proteomics_IJMS_Figures.pdf]

Proteomic analysis of ARID1A-deficient ovarian clear cell carcinoma cells reveals differential mitochondria ETC subunit abundances and targetable mitochondrial pathways

Jesenia M. Perez[1], Joohyun Ryu[2], Hannah Khan[3], Mihir Shetty[3], Emma Parker[4], Padraig D'Arcy[5], Shijia Zhu[2], Martina Bazzaro[3,5], and Stefani N. Thomas[2]*

[1]Microbiology, Immunology, and Cancer Biology Graduate Program, University of Minnesota School of Medicine, Minneapolis, MN, USA

[2]Department of Laboratory Medicine and Pathology, University of Minnesota School of Medicine, Minneapolis, MN, USA

[3]Masonic Cancer Center and Department of Obstetrics, Gynecology and Women's Health, University of Minnesota, Minneapolis, MN, USA

[4]School of Biological, Health and Sports Sciences, Technological University Dublin, Dublin, Ireland

[5]Department of Biomedical and Clinical Sciences (BKV), Linköping University, 58183 Linköping, Sweden.

SUPPLEMENTAL FIGURES

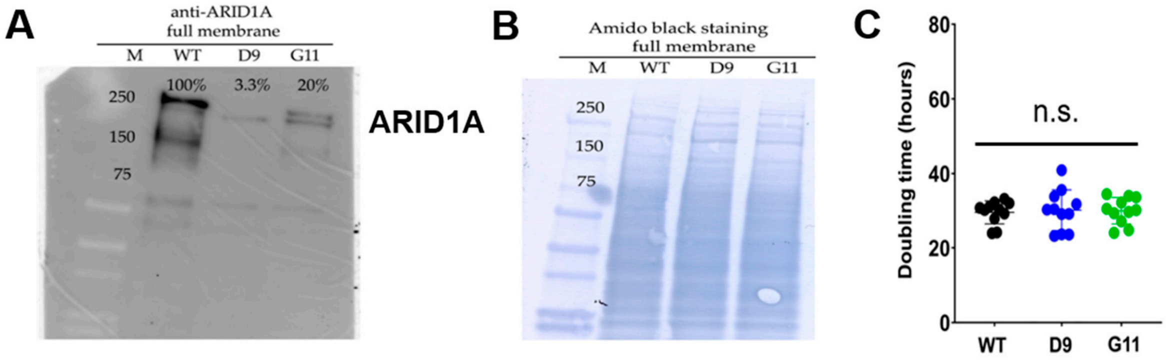

Figure S1. Characterization of ARID1A knockout (KO) clones. A) Representative Western blot analysis of ARID1A expression levels in cell lysates of WT, D9, and G11 ARID1A clones of the OCCC-derived cell line, RMG1. Percentage indicates the relative amount of ARID1A in the WT (100%), D9 (3.3%), and G11 (20%) clones. M = molecular weight marker. ARID1A molecular weight is 250 kDa. B) Amido black-stained membrane demonstrating equal protein loading. C) Average cell doubling time of WT, D9, and G11 clones is not significantly different following 12 passages. Error bars indicate mean ±S.D. n.s., not significant. Student's t-test.

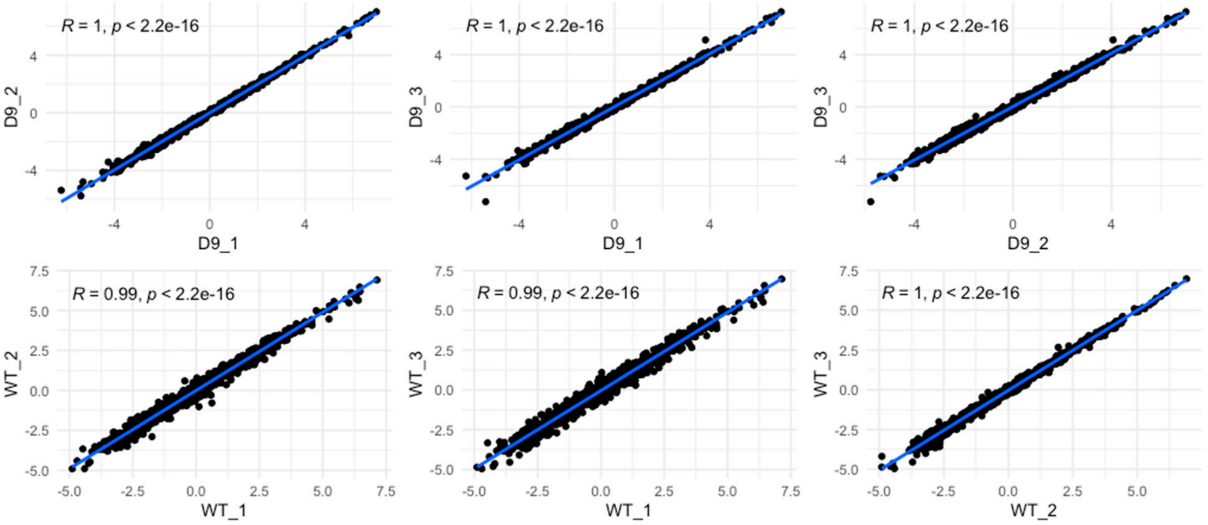

Figure S2. Correlation plots of quantified proteins across biological samples. D9, ARID1A knockout; WT, ARID1A wild-type. R, Pearson correlation coefficient.

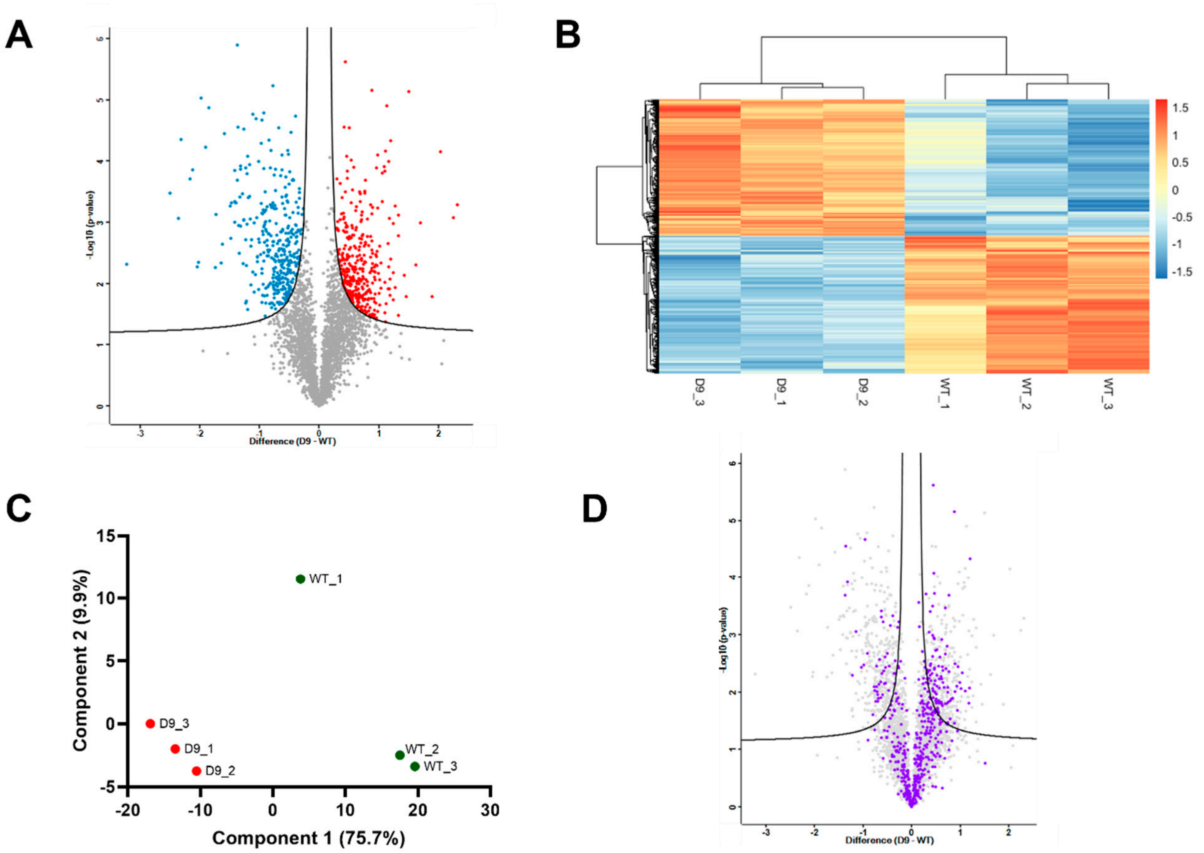

Figure S3. Differentially abundant proteins associated with loss of ARID1A expression. A) Distribution of proteins quantified across all samples with no missing values (n=3,316). Significantly dysregulated proteins are indicated in red (up-regulated proteins) and blue (down-regulated proteins). Permutation-based false discovery rate (FDR, $p < 0.01$, s0 = 0.0778) estimation is visualized with black hyperbolic curves. B) z-score-based hierarchical clustering map of the 701 differentially expressed proteins across all D9 and WT samples. C) Principal Component Analysis (PCA). D) Relative abundance of differentially expressed proteins, with mitochondrial proteins highlighted in purple.
